# Clinical Factors and the Outcome of Treatment with Methotrexate in Rheumatoid Arthritis: Role of Rheumatoid Factor, Erosive Disease and High Level of Erythrocyte Sedimentation Rate

**DOI:** 10.3390/jcm11206078

**Published:** 2022-10-14

**Authors:** Edyta Majorczyk, Małgorzata Mazurek-Mochol, Andrzej Pawlik, Piotr Kuśnierczyk

**Affiliations:** 1 Department of Physiotherapy, Faculty of Physical Education and Physiotherapy, Opole University of Technology, 45-758 Opole, Poland; 2 Department of Periodontology, Pomeranian University of Medicine, 70-111 Szczecin, Poland; 3 Department of Physilogy, Pomeranian University of Medicine, 70-111 Szczecin, Poland; 4 Laboratory of Immunogenetics and Tissue Immunology, Ludwik Hirszfeld Institute of Immunology and Experimental Therapy, Polish Academy of Sciences, 53-114 Wrocław, Poland

**Keywords:** rheumatoid arthritis, treatment response, methotrexate, biomarkers

## Abstract

To identify the clinical factors predicting the outcome of treatment with methotrexate in rheumatoid arthritis, we examined 312 patients (253 females, 59 males) with rheumatoid arthritis diagnosed according to the criteria of the American College of Rheumatology. All patients included in this analysis began treatment with a regimen of oral MTX 7.5 mg weekly, with the dosage increasing to 15 mg weekly after 4 weeks, in combination with folic acid (1 mg daily). Good responders were defined as patients who had a DAS28 of ≤2.4 at 6 months (patients with remission of disease symptoms). Poor responders were defined as patients who had a DAS28 of >2.4. In this study, we analyzed the association between clinical parameters such as sex of patients, age of patients, age at disease onset, disease duration, rheumatoid factor, anti-CCP antibodies, ESR values, presence of joints erosions, presence of extra-articular manifestations and the response to MTX in RA patients. Multivariate logistic regression analysis showed four independent factors significantly associated with good response to MTX treatment: older age at disease onset, low ESR, no erosive disease and negative RF. The results of our study suggest that a younger age at disease onset, the presence of RF, erosive disease, as well as a high level of ESR are associated with worse response to MTX therapy.

## 1. Introduction

Rheumatoid arthritis (RA) is a common autoimmune disease in which treatment is addressed to prevent joint deformities, impaired function and disability. It is mainly based on disease-modifying antirheumatic drugs (DMARDs) in order to reduce disease activity as well as obtain disease remission, especially as an early intervention. In clinical practice, among conventional DMARDs, methotrexate (MTX) is still internationally recommended as a gold standard for RA therapy [1,2,3].

MTX is a folate antagonist still used in the therapy of malignant disorders as an inhibitor of the synthesis of nucleic acids (DNA and RNA) and proteins via binding to dihydrofolate reductase. Low doses of MTX were introduced for the treatment of RA and other autoimmune diseases because of its presumed anti-proliferative, immunosuppressive and anti-inflammatory properties [4,5], including downregulation of production of proinflammatory cytokines, suppression of lymphocyte proliferation, neutrophil chemotaxis and adherence, and a reduction in serum immunoglobulin level [6,7].

Rapid clinical remission of the disease after MTX treatment suggests that the anti-inflammatory elements of the mechanisms of MTX action play a much larger part in RA treatment than the anti-proliferative ones [7]. In comparison to other DMARDs, MTX monotherapy is more effective in a reduction in disease activity, patients’ disability and in cessation of the progression of radiographic damages [8,9]. However, the mechanism by which MTX at a low dose (up to 25 mg/week) modulates a course of RA is only partly understood. On the other hand, despite the low dose, using MTX creates toxicity and adverse effects and some patients have to discontinue treatment. Moreover, a significant proportion of patients stop the therapy because of its inefficiency (about 30–40% of RA patients treated with MTX are qualified as poor responders). Nevertheless, the MTX for RA therapy is a drug with the best efficiency/toxicity ratio [10].

The identification of patients who are predicted to respond worse to the MTX (due to toxicity or/and inefficiency) is still difficult. Among agents related to this phenomenon, female sex, longer disease duration, younger age at onset, severe disease progression as well as genetic backgrounds are commonly suggested [10,11,12,13,14]. However, studies examining disease activity parameters and clinical factors as predictors of MTX treatment efficiency are inconsistent [7]. Some studies suggest that higher disease activity parameters are associated with poorer response to MTX therapy, while other studies indicate better efficacy of MTX therapy in these patients.

For this reason, in our study, we evaluated whether some clinical factors may be used to predict the outcome of treatment with methotrexate in patients with rheumatoid arthritis.

## 2. Materials and Methods

### 2.1. Study Population

In this retrospective study, we examined 312 patients (253 females, 59 males) with RA diagnosed according to an American College of Rheumatology/European League Against Rheumatism collaborative initiative 2010 rheumatoid arthritis classification criteria [15]. Patients were treated in the Department of Rheumatology, County Hospital in Szczecin, Poland. All subjects were Caucasian from the Pomeranian region of Poland. At the beginning of treatment, all patients were hospitalized at the Department of Rheumatology, where they underwent all diagnostic procedures. They then attended the Department of Rheumatology for follow-up examinations to assess the effectiveness of the treatment. The subjects were evaluated by a rheumatologist.

The subjects enrolled in the study underwent routine biochemical blood analysis, and when required, assays for anti-cardiolipin antibodies, anti-nuclear antibodies, and immunological complexes. X-rays of the chest, hands, and feet were obtained in all patients and, when required, radiographs of other joints. These were interpreted by two expert radiologists. The evaluation of the subjects included physical examination, with a particular focus on the pattern of joint involvement and the occurrence of extra-articular features, such as vasculitis, sicca syndrome, and amyloidosis, as well as laboratory features, such as rheumatoid factor (RF) and anti-cyclic citrullinated peptide antibodies (anti-CCP), screened with the use of respective ELISA kits. Amyloidosis was diagnosed by histomorphology (skin and bowel or duodenum biopsy), vasculitis by histomorphology (skin biopsy) and angiogram. 

All patients included in this analysis began treatment with a regimen of oral MTX 7.5 mg weekly, with the dosage increasing to 15 mg weekly after 4 weeks, in combination with folic acid (1 mg daily). The baseline characteristics of patients with rheumatoid arthritis are shown in Table 1. The study was approved by the Ethics Committee of Pomeranian Medical University, Szczecin, Poland (KB-0012/40/14), and written informed consent was obtained from all subjects.

### 2.2. Evaluation of Clinical Efficacy 

In the study group, 179 (57.4%) patients were classified as good responders, while 133 (42.6%) were classified as poor responders. Good responders were defined as patients who were receiving MTX and had a disease activity score based on 28 joint counts (DAS28) [16] of ≤2.4 at 6 months (patients with remission of disease symptoms). Poor responders were defined as patients who were receiving MTX and had a DAS28 of >2.4 [17].

### 2.3. Statistical Analysis

The comparison of clinical parameters between good responders and poor responders was assessed using Fisher exact test or Mann–Whitney U test. To investigate which clinical variables are independent factors affecting the probability of good response to MTX treatment multivariate logistic regression model was used after the logarithmic transformation of quantitative independent variables with non-normal distribution. The odds ratio (OR) with a 95% confidence interval (95%CI) was computed as a measure of effect size. Arithmetic mean and standard deviation were calculated for continuous variables. Results were regarded as statistically significant at *p* < 0.05.

## 3. Results

In the first step of our study, we analyzed the association between clinical parameters (sex of patients, age of patients, age at disease onset, disease duration, rheumatoid factor, anti-CCP antibodies, erythrocyte sedimentation rate (ESR) values, presence of joints erosions, presence of extra-articular manifestations) and the response to MTX in RA patients.

Under therapy with MTX, a remission of RA symptoms was achieved in 57.4% of the total group of RA patients (61% in men and in 56.5% in women). There was no statistically significant difference between men and women. There were also no statistically significant differences in relation to the age of good responders and poor responders as well as disease duration. The age at disease onset was statistically significantly higher in good responders (Table 2). The baseline values of erythrocyte sedimentation rate (ESR) in the good responder group were statistically significantly lower than in the poor responder group (27.3 and 58.1 mm/h, respectively; *p* < 0.0001) (Table 2).

The remission of disease activity was more frequently observed in RF-negative than in RF-positive patients. This difference was statistically significant (Table 3). The same trend was achieved for erosive disease and extra-articular manifestations: a probability of remission of RA symptoms was statistically significantly higher in patients without joint erosions than in those with bone erosion as well as in patients without extra-articular manifestations than in those with extra-articular manifestations. In contrast, the presence of anti-CCP antibodies had any impact on methotrexate therapy efficacy (Table 3).

In accordance with the above, patients negative for both RF, bone erosion and extra-articular manifestations achieved remission almost 3 times more frequently than subjects positive for these two parameters (88.2% vs. 29.6% of good responders) (Table 3). 

In the next step of our study, multivariate logistic regression analysis was performed to see which clinical parameters are independent factors affecting the efficacy of MTX treatment.

Multivariate logistic regression analysis showed four independent factors significantly associated with good response to MTX treatment: older age at disease onset, low ESR, no erosive disease and negative RF (Table 4).

## 4. Discussion

MTX is one of the most commonly used DMARDs for the treatment of RA. This drug slows the progression of RA and contributes to maintaining long-term remission of RA symptoms. MTX is generally well tolerated by patients and does not cause severe side effects. Previous observations have shown that the effectiveness of MTX therapy varies among patients. Therefore, various factors are being sought that can affect the efficacy of therapy and be helpful in the proper choice of drug and its dosage regimen. Among these factors, genetic factors are a large group, and clinical parameters that can affect the effectiveness of therapy are also considered. In this study, we analyzed the association between basic clinical parameters and the efficacy of MTX therapy in RA patients. Among the parameters considered were: the sex of patients, age of patients, age at disease onset, disease duration, rheumatoid factor, anti-CCP antibodies, ESR values, presence of joint erosions, and presence of extra-articular manifestations. The performed multivariate logistic regression analysis indicated that a younger age at disease onset, the presence of RF, erosive disease as well as a high level of ESR were associated with worse response to MTX therapy. Our results suggest that patients diagnosed with RA at an earlier age, with baseline high disease activity, with rheumatoid factor, and with the presence of joint erosions, are more likely to have an ineffective response to MTX. In these patients, consideration should be given to possibly implementing another drug, adding a second drug to MTX, or possibly administering MTX at a higher dose. Previous studies suggest that subcutaneous MTX may be considered in patients who have demonstrated the ineffectiveness of orally administered MTX [18]. To date, there are many studies evaluating the relationship between clinical parameters and response to MTX, but their results are often contradictory. Some studies suggest that baseline higher disease activity parameters are associated with a poorer response to MTX, while other studies show an association of baseline higher disease activity parameters with a better response to MTX [10,11,12,13,14].

Generally, it seems that disease severity impacts therapy responsiveness, and patients with the more active disorder as well as those longer affected with RA are more likely to respond inefficiently to therapy. It is known that severe disease progression could be predicted with RF and anti-CCP positivity at screening [12,19,20]. Although “the role of RF in RA pathogenesis remains a conjecture” and anti-citrullinated protein antibodies are more specific than RF for RA, our results suggest a predictive value of RF rather than anti-CCP for the MTX treatment outcome [21]. This finding contradicts previous results showing that RF is not a predictor of the effectiveness of MTX as well as other DMARDs therapy [22]. Nevertheless, it seems that MTX does not affect autoantibody levels, which are reduced by DMARDs targeting the adaptive immune response and immunosuppression [23,24,25,26]. On the other hand, in our study, the percentages of responders in anti-CCP-positive and -negative patients did not significantly differ, despite previous literature suggestions that achievement of sustained drug-free remission (DFR) is more likely in patients who are negative for this autoantibody [20,27].

It is also suggested that DMARD-related DFR is associated with other predictive factors, separately considered: swollen/tender joint count, patient and physician global assessment and the inflammatory variables (CRP and ESR) [28,29,30]. It is known that high CRP levels, high ESR, or persistent disease activity were associated with higher radiographic progression in the group taking MTX alone in comparison with patients receiving both MTX and infliximab [31]. On the other hand, our study confirms that a level of ESR correlates even with the clinical response to MTX [32].

Nevertheless, the MTX treatment outcome could differ in patients with the same disease severity (measured by ESR level, presence of ED and/or RF). Our results show that younger age at disease diagnosis is associated with poorer MTX therapy efficiency. This finding confirms the younger age at onset as a predictor of worse response to MTX [33]. No link, however, between the duration of disease and response to MTX was found which is in accordance with some previous studies [32,34].

Poorer response to treatment related to increased disease activity in women than in men is also found in the QUEST-RA study [35]. It is in accordance with the majority of studies which observed a better response to treatment (with MTX alone as well as with MTX combined with anti-TNF agents) in men than in women both in early and established RA. Moreover, female patients were more likely to stop therapy for adverse events [32,33,34,36]. These differences could be explained as a result of variations in the pathogenesis of early RA in men and women, differences in drug metabolism or gender-related differences in pain perception and reporting affecting disease activity scores [37,38]. In our study, we also observed a higher frequency of good responders among men; however, this difference did not reach statistical significance.

The aforementioned differences with regard to the clinical factors predicting the outcome of treatment with methotrexate in rheumatoid arthritis may be caused by the various definitions of RA remission. Sokka et al. compared the different definitions of remission in a large multinational cross-sectional cohort of patients with RA [39]. The database was analyzed according to the following definitions of remission: American College of Rheumatology (ACR) definition, Disease Activity Score in 28 joints (DAS28), Clinical Disease Activity Index (CDAI), and clinical remission assessed using 42 and 28 joints patient self-report. The authors conclude that the use of different definitions of RA remission leads to different results with regard to remission rates, with considerable variation among countries and between sexes. Reported remission rates in clinical trials and clinical studies have to be interpreted in light of the definition of remission that has been used. 

In summary, we attempted to predict good clinical response as defined by a DAS28 ≤2.4 after 6 months of MTX treatment. In this model RF, erosive disease, higher level of ESR and younger age at RA onset were associated with significantly poorer response to MTX therapy. These findings suggest that disease functional class and disease activity have effects on the likelihood of patient response to treatment. In conclusion, we identified a number of clinical and laboratory parameters that are independent predictors of clinical remission in RA patients. To better understand the complex prognostic pathways or processes of RA, these relationships need to be further investigated in explanatory prognostic studies.

## 5. Conclusions

The results of our study suggest that a younger age at disease onset, the presence of RF, erosive disease, as well as a high level of ESR are associated with worse response to MTX therapy.

## Figures and Tables

**Table 1:** Baseline clinical characteristics of patients with rheumatoid arthritis.

Parameters	
Female sex	253
Male sex	59
Age (years)	58.0 ± 12.7
Age at disease onset (years)	48.0 ± 13.1
Disease duration (years)	10.0 ± 8.8
ESR (mm/h)	40.42 ± 24.61
RF+	208
CCP+	193
Vasculitis	56
Sicca syndrome	24
Amyloidosis	14

ESR—erythrocyte sedimentation rate, RF—rheumatoid factor, CCP-anti-cyclic citrullinated peptide antibodies

**Table 2:** Clinical characteristics in good responders and poor responders.

Groups	Age (Mean ± SD)	Disease Duration (Mean ± SD)	Age at Onset (Mean ± SD)	ESR (mm/h) (Mean ± SD)
Good responders	59.4 ± 12.4	9.0 ± 7.3	50.4 ± 12.3	27.3 ± 18.75
Poor responders	56.1 ± 12.9	11.3 ± 10.0	44.9 ± 13.5	58.1 ± 39.47
*p*	0.32	0.27	0.0001	<0.0001

*p*—Good responders vs. poor responders, Mann–Whitney U test. Age at onset—the age of the patient at which the first symptoms of the disease were diagnosed. Disease duration—time from the diagnosis of the first symptoms of the disease to the inclusion of the patient in the study. ESR—erythrocyte sedimentation rate.

**Table 3:** Efficiency of MTX therapy depending on clinical parameters.

Remission (%)	Patients Subgroup											
	Female Sex	Male Sex	RF+	RF−	ED+	ED−	CCP+	CCP−	Extra+	Limited	RF+	RF−
										to	ED+	ED−
										Joints	Extra+	Extra−
	n-253	n-59	n-208	n-104	n-231	n-81	n-193	n-119	n-94	n-218	n-54	n-34
Good response	56.5	61.0	47.1	77.9	49.8	77.8	53.9	57.1	44.7	62.8	29.6	88.2
Poor response	43.5	39.0	52.9	22.1	50.2	22.3	46.1	42.9	55.3	37.2	70.4	11.8
*p* value *^#^*	0.65		<0.0001		<0.0001		0.61		0.003		<0.0001	
OR	0.83		0.25		0.28		0.87		0.48		0.06	
95%CI	0.47–1.48		0.15–0.43		0.16–0.51		0.55–1.39		0.29–0.78		0.02–0.19	

ED—erosive disease; RF—rheumatoid factor; Extra—extra-articular manifestations; OR, odds ratio; CI—confidence intervals, *p ^#^*—Fisher exact test.

**Table 4:** Multivariate logistic regression model predicting good response to methotrexate treatment.

Independent Variables	OR	95%CI	*p*-Value
Age at disease diagnosis	1.055	1.023–1.086	0.0007
Log(ESR) *	0.020	0.009–0.056	<0.0001
ED	0.417	0.163–0.974	0.042
RF	0.316	0.148–0.736	0.006

* Natural logarithm of ESR. ED—erosive disease; RF—rheumatoid factor, ESR—erythrocyte sedimentation rate.

## Data Availability

Not applicable.
